# Prevalence of drug resistance mutations in HIV-1 protease gene from North India

**DOI:** 10.1186/1471-2334-12-S1-P84

**Published:** 2012-05-04

**Authors:** Mohd Azam, Abida Malik, Meher Rizvi, Supriya Singh, Hanu Ram, Megha Singhal, Poonam Gupta

**Affiliations:** 1 Department of Microbiology, J. N. Medical College, Aligarh Muslim University, Aligarh, India; 2 Division of Biochemistry & Biotechnology, National Centre for Disease Control, New Delhi, India

## Background

Success of combination ART therapy in treating HIV infection is hampered due to emergence of drug resistant mutations in polymerase gene. The present study was conducted to find out the prevalence of drug resistance-conferring mutations in *protease* gene in HIV-1 infected patients.

## Methodology

CD4 cells estimation was done in all patients. The *protease* gene was amplified from pro-viral DNA by nested PCR and then sequenced. Mutational analysis and subtyping were done by using Stanford database and REGA HIV-1 subtyping tool, respectively.

## Results

Among 35 patients, there were 17 drug naïve and 18 first line drugs experienced HIV-1 infected patients (22 males & 13 females; mean age: 35.95 years; mean CD4 cells: 216.4cells/mm^3^). Majority of our patients showed mutations at T12S/T (82.85%), K14R (40%), I15V (71.42%), L19I/T/V/M (97.14%), M36I (71.42%), R41K (88.57%), L63P (65.71%), H63K (100%) and L89M (74.28%) positions in both group of patients while other mutations were at positions 35, 37, 45, 60, 62, 77, and 82 in few cases. Interestingly, one first line drug experienced patient showed major DR mutations at D30N and M46I positions. Majority (94.28%) was belonging to subtype C and 2 patients were belonging to subtypes A (A1).

## Conclusion

HIV-1 subtype C predominates in northern India followed by subtype A. Major DR mutation M46I are suggested to confer low levels of resistance to ATV, FPV, IDV, LPV, NFV and TPV.D30N confers resistance only to NFV. Resistance testing in HIV-1 infected patients should be performed before the initiation of therapy for better therapeutic outcome.

